# 4D MRI-based wall shear stress quantification in the carotid bifurcation: a validation study in volunteers using computational fluid dynamics

**DOI:** 10.1186/1532-429X-16-S1-P162

**Published:** 2014-01-16

**Authors:** Wouter V Potters, Merih Cibis, Henk A Marquering, Ed vanBavel, Frank Gijsen, Jolanda J Wentzel, Aart J Nederveen

**Affiliations:** 1 Radiology, Academic Medical Center, Amsterdam, Netherlands; 2 Biomedical Engineering, Erasmus Medical Center, Rotterdam, Netherlands; 3 Biomedical Engineering & Physics, Academic Medical Center, Amsterdam, Netherlands

## Background

Remodeling of the vessel wall is associated with wall shear stress (WSS) magnitude (Malek, 1999). It has been suggested that local low or high WSS may respectively promote or prevent atherosclerotic lesions in the carotid vessel wall. The current procedure for WSS quantification is computational fluid dynamics (CFD), which is time-consuming and not always patient-specific. In the past years, methods that derive WSS directly from MRI 4D flow measurements have been developed. These allow patient-specific WSS quantifications while using less computational power and time. We tested to what extent MRI-based WSS maps match the patient-specific CFD-base maps. To accomplish this, a pipeline for simultaneous MRI- and CFD-based WSS calculations was developed and applied to the carotid arteries of 6 healthy volunteers.

## Methods

After providing informed consent, six healthy volunteers were scanned with a 3T MRI scanner (Philips Healthcare, the Netherlands) using a dedicated 8-channel carotid coil. 4D flow MRI data were acquired: spatial resolution 0.63 mm, temporal resolution ~140 ms, TE/TR/flip angle 3.1 ms/6.7 ms/15 degrees, scan duration 25 minutes. We manually segmented the carotid bifurcation in the 4D flow MRI images and converted this segmentation to a volume mesh for CFD calculations. Time-resolved CFD simulations were performed with a characteristic flow waveform, which was matched to measured in- and outlet flow rates, resulting in CFD-based WSS values. For the MRI-based WSS, we directly calculated shear rates for the entire vessel wall by fitting a smoothing spline to the measured velocities along the inward normal of each point on the vessel wall. (Potters 2012, van Ooij 2013) The CFD and MRI based calculations took ~15 hours and ~15 minutes respectively. The WSS in CFD and MRI was compared by means of overlap between low, median and high WSS tertiles.

## Results

**Figure 1:**
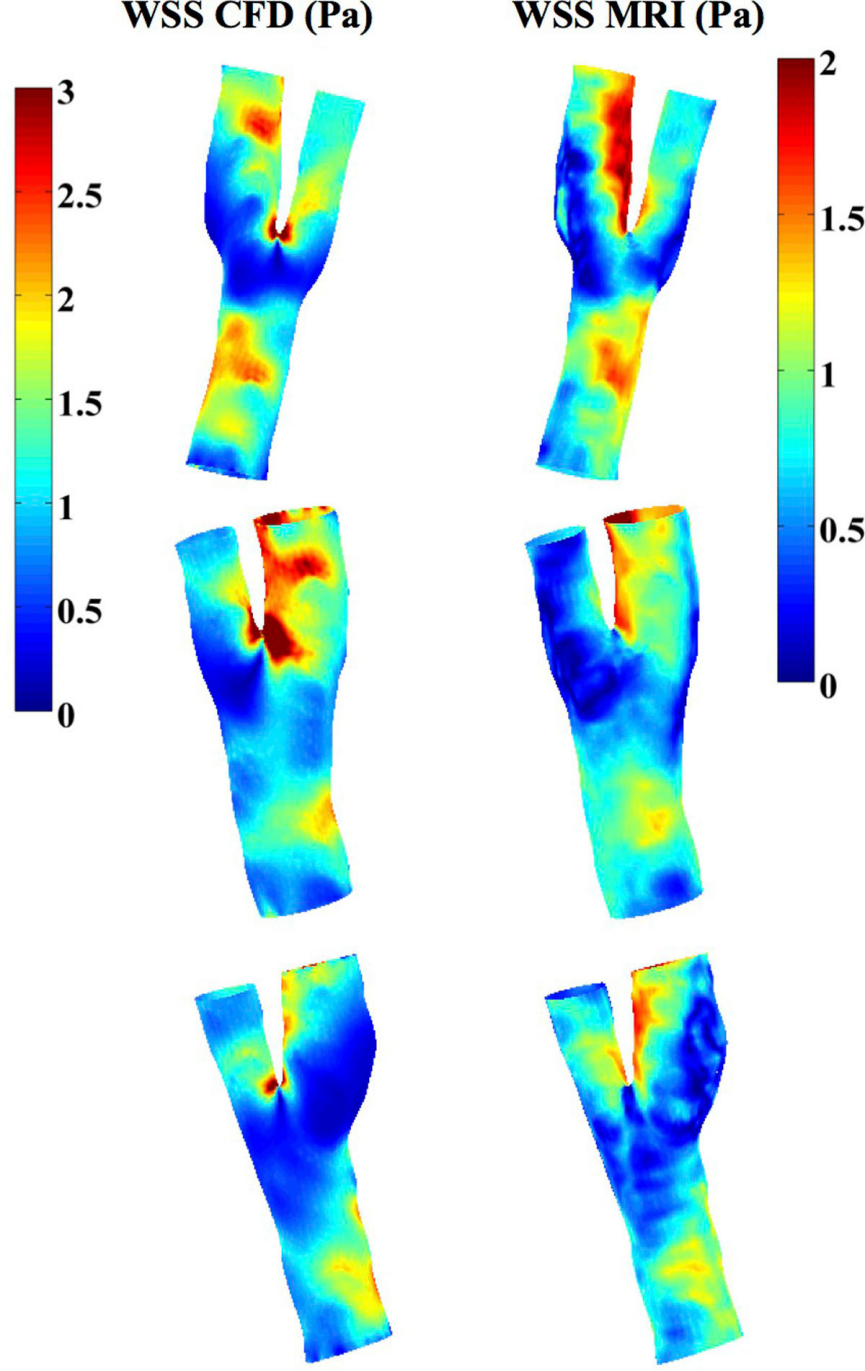
**WSS magnitude [Pa] distribution at diastole for 3 carotids calculated using CFD (left side, 0-3 Pa) and directly from 4D flow MRI (right side, 0-2 Pa)**.

**Figure 2:**
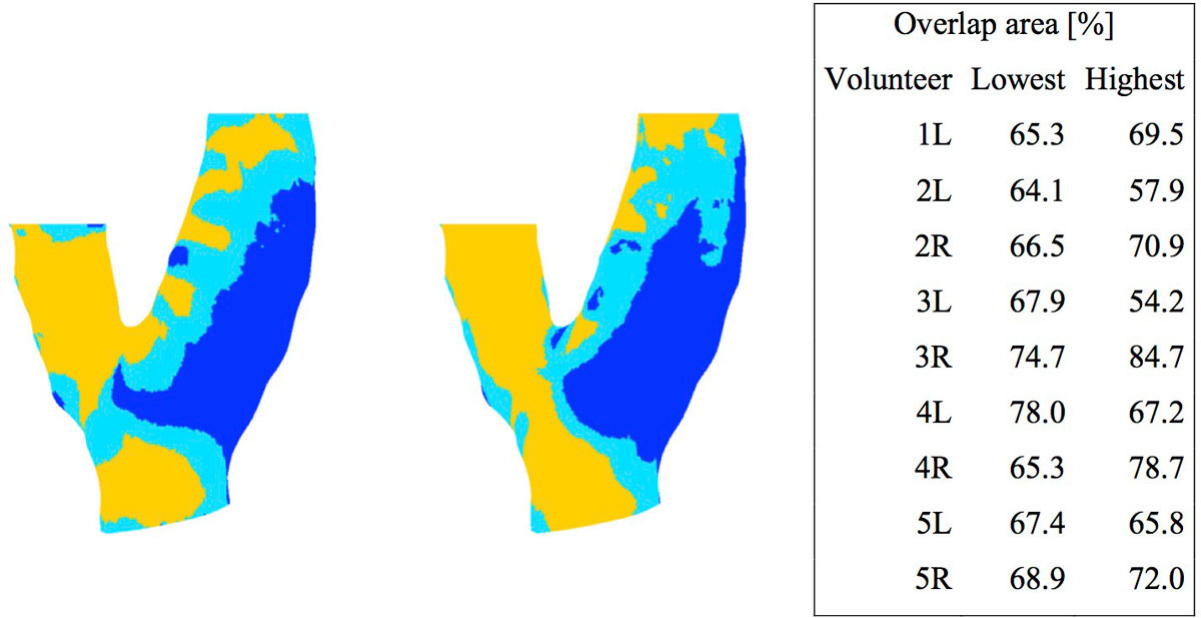
**WSS distribution of example carotid bifurcation in 3 bins**. Lowest (blue), middle (light blue) and highest WSS tertiles as calculated by CFD (left) and calculated using 4D flow MRI (right). The table on the right shows the overlap areas for the lowest and highest WSS tertile for all volunteers.

The locations of low, median and high WSS matched well when estimated from MRI versus CFD (Figure 1). Overlap between modalities was 70% (± 7%, averaged over 5 volunteers and low and high tertile) (Figure 2). The systematic larger CFD-based WSS magnitudes (factor 1.5-2) compared to the MRI-based WSS could be explained by the difference in resolution (Stalder 2008, Petersson 2012).

## Conclusions

Although WSS was underestimated with 4D flow MRI, both the CFD and the 4D flow MRI based calculations resulted in similar low and high WSS patterns. More work is needed to extend this work to also compare the direction of the WSS.

## Funding

Dutch Technology Foundation STW [Carisma 11629].

